# Security and cost comparison of INR self-testing and conventional hospital INR testing in patients with mechanical heart valve replacement

**DOI:** 10.1186/s13019-015-0205-1

**Published:** 2015-01-16

**Authors:** Qiu-lin Chen, Li Dong, Yi-jun Dong, Shu-lin Zhao, Bo Fu, Yu-qing Wang, Hong Jiang

**Affiliations:** 1 Department of Cardiovascular Surgery, West China Hospital, Sichuan University, Chengdu, 610041 P.R. China; 2 Department of Oncology, West China Hospital, Sichuan University, Chengdu, 610041 P.R. China; 3 Department of Clinical Laboratory, West China Hospital, Sichuan University, Chengdu, 610041 P.R. China

**Keywords:** Heart valve replacement, Anticoagulation therapy, Portable coagulometer, Self-testing, Cost

## Abstract

**Background:**

International normalized ratio (INR) self-testing can improve the management of anticoagulation therapy with warfarin for the patients following mechanical heart valve replacement. Several reviews and studies have demonstrated self-management as an option to improve patient’s outcome considerably after mechanical heart valve replacement. We sought to analyze the security, economy and discuss the prospect of self-testing of anticoagulation therapy in patients following mechanical heart valve replacement in China, and evaluate the accuracy and stability of CoaguChek XS portable INR-testing device.

**Methods:**

This was a prospective self-controlled clinical study conducted with 526 patients receiving oral warfarin anticoagulation therapy after mechanical heart valve replacement in the period of Mar.1, 2012 – Nov.1, 2012 in Cardiovascular Surgery Department of West China Hospital of Sichuan University. The same patient performed INR testing with CoaguChek XS portable coagulometer (group1) and central lab (group 2) in parallel. The follow-up time was 6 months. Meanwhile, a questionnaire was handed out to survey the expenses required for the re-examination visits to the hospital, time, and anticoagulation complications.

**Results:**

No severe anticoagulation complications occurred in all the patients. No significant difference of the INR results were observed between group 1 and group 2, they showed significant relevance, r = 0.953(p < 0.05). Compared with the conventional method of INR testing in hospital, the portable coagulometer is convenient, quick and less traumatic. Self-testing of anticoagulation therapy reduced the cost and the time required for re-examination.

**Conclusions:**

Results of CoaguChek XS monitor are precise and have a good consistency and stability as compared with traditional laboratory testing. For the patients receiving anticoagulation therapy after mechanical heart valve replacement, the self-testing of anticoagulation therapy with portable coagulometer is a safe choice, and it has a promising future application in China.

## Background

As one of the most common adult cardiac surgeries,there has been over 200,000 cases of cardiac valve replacement surgeries in China [1]. The mean age of patients with valvular diseases in China are young and over 98% of them received cardiac mechanical valves [2]. After mechanical valve replacement surgery, the patient must adhere to a life long anticoagulation therapy,and the limited therapeutic index of warfarin requires regular check of the INR to adjust its anti-coagulation intensity to improve the quality of anticoagulation management and avoid complications with either higher or lower blood-coagulation level [3–5].

Traditional method to monitor anticoagulation requires the patients to visit professional medical institutions regularly. Plasma after anticoagulation centrifugation is drawn from venous blood and sent to lab for testing . Doctor will instruct them to adjust the dosage of orally administered warfarin. It has the advantage of accurate measurement which gives the patient professional guidance on anticoagulation after testing. However, such re-examination visits are costly and time-consuming. Many of them failed to receive re-examination on a regular basis. It was shown in a study, the incidence of follow-up re-examination of coagulation functions received after more than half a year after surgery account for 38% of the total [6]. Excessively low frequency of monitoring may obviously affect the anticoagulation therapy management, increasing the incidence of complications, and therefore, greatly increase the risks of anticoagulation therapy [7,8].

Self-testing of INR using Portable point-of-care (POC) coagulometers has been used in clinical application for nearly 20 years in other countries. The portable coagulometer features easy manipulation, fast and accurate measurement. The patients needing oral warfarin anticoagulation in developed western countries have started to use portable coagulometer for self-testing and self-management [9–14]. In China, reports and applications are limited to the analysis of lab data of portable coagulometer [14,15]. There are no clinical application reports or evaluation of economy based on massive samples, or any reports on self-testing of anticoagulation therapy. Presently in China, although most patients have accepted low-intensity anticoagulant therapy after cardiac valve replacement, the incidence rates of relevant complications still remains high, mainly due to the fact that the patients conduct anticoagulation testing with a low frequency and fail to regularly re-examine or timely adjust the dosage of warfarin which results in complications. Overseas literatures indicate that self-monitoring may increase patient’s monitoring frequency and reduce the incidence rate of complications [16,17]. Therefore, it is compulsory to conduct clinical studies on self-testing of anticoagulation therapy after mechanical heart valve replacement surgery. The purpose of this study is to analyze the security, economy and discuss the prospect of self-testing of anticoagulation therapy in patients following mechanical heart valve replacement in China, and evaluates the accuracy and stability of CoaguChek XS portable INR-testing device.

## Materials and methods

The design and implementation of this study has been conducted based on the approval of West China Hospital of Sichuan University Clinical Test Ethics Committee and adhered to the tenets of the Declaration of Helsinki. Additionally, the written informed consent was obtained from the patients.

### Patients

We selected the 526 consecutive patients conducting anticoagulation therapy follow-up from Mar.1, 2012 – Nov. 1, 2012, after receiving mechanical valve replacement surgery in the Cardiovascular Surgery Department of West China Hospital of Sichuan University. In this study, we used the average exchange rate in 2012 of the RMB (Chinese Yuan) against the U.S dollar for 6.3125. The inclusion criteria of the patients were:

- Receiving only oral warfarin for anticoagulation therapy after receiving mechanical valve replacement surgery.
- Without history of administering other medicines affecting anti-coagulation functions.
- Without severe coagulation diseases.
- Without drug and alcohol abuse.
- Having a good compliance.
- Patients or their actual caregivers having the ability to fulfill self-testing criteria recommended by professional bodies.

### Methods

The follow-up time is 6 months. Low-intensity anticoagulation therapy is accepted by the patients after mechanical valve replacement. The INR target value of anticoagulation therapy: AVR 1.5-1.8, MVR and DVR1.8-2.0, TVR including other valve replacement 2.0-2.5. According to the current tradition, the monitoring frequency is 1 week/time within one month after the valve replacement surgery, 2 weeks/time within 1–3 months after the valve replacement surgery, and 1–3 months/time after 3 months. All these patients attend the hospital outpatient anticoagulation clinic for INR testing. Professional physicians will adjust the dosage of orally administered warfarin.

To re-examine coagulation functions of these patients, the traditional central lab testing methods and self-testing methods are used in parallel to check and record the INR values of the patients. In the former case, peripheral venous blood of about 3 cm^3^ is collected by the laboratory staff, and added into a standard sodium citrate anticoagulation blood collection tube, and tested with STAGO STAR automatic coagulometer after plasma separation. In the latter case, about 0.01 cm^3^ blood at the end of capillary of finger is collected by the patients themselves or their actual caregivers, and added into corresponding area on the dry reaction test strip, and tested with CoaguChek XS portable coagulometer.

For the purpose of analyzing the costs of the two testing methods, the general conditions of the patients, expenses (registration fee, testing fee, transportation fee and accommodation, etc.) and time (transportation and accommodation time + time of registration, testing, diagnosis, queuing and waiting, etc.) for the re-examination visits to the hospital were investigated in the form of questionnaire. Meanwhile, survey the anticoagulation complications. In the study, the expenses and time for each re-examination visit varies with the geographic location of the patients, so patients from different localities were grouped into Chengdu Group (Local group), Sichuan Group (except Chengdu) (Within-Sichuan Group), and Other Locality Group (Outside-Sichuan Group).

In the analysis using SPSS 18.0 statistic software, the test results were expressed in average ± standard deviation ($\overline{x}\pm s$), and p values less than 0.05 were considered statistically significant. The linear regression and correlation of the two methods were analyzed and scatter diagram and regression line of the two were plotted; meanwhile, Bland-Altman analysis was adopted to plot scatter diagram with the deviation and average of INR measured respectively with the two methods as subject of study to obtain 1imits of agreement and compare the correlation between the standard deviation of INR difference and the imprecision of INR values measured respectively with the two instruments, and evaluate the consistency of INR values. Perform comparison study on the economic indicators such as expenses and time with two anticoagulation monitoring methods for patients of various localities.

## Results

### Basic characteristics of the study object

The cases including 177 cases of male patients (33.7%) and 349 cases of female patients (66.3%), with age of 20–76 (48.75 ± 10.29). There are 263 cases of mitral valve replacement (MVR) (50.0%), 91 cases (17.3%) of aorta valve replacement (AVR), 147 cases (27.9%) of mitral valve and aorta valve replacement (DVR), 12 cases (2.3%) of tricuspid valve replacement surgery (TVR), 9 cases (1.7%) of mitral valve and tricuspid valve replacement surgery (MVR + TVR), 4 cases(0.8%) of mitral valve and aorta valve with tricuspid valve replacement surgery (DVR + TVR). All the patients use St. Jude Medical bileaflet Mechanical Heart Valve. Patients from different localities were grouped into three groups. Among them, 141 cases (26.8%) belong to Local Group; 368 cases (70.0%) belong to Within-Sichuan Group; and 17 cases (3.2%) belong to Outside-Sichuan Group (Table 1).

**Table 1:** **Basic characteristics of the study object**

		Headcount (Persons)	Percentage (%)
Total		526	100.0
Gender	Male	177	33.7
	Female	276	66.3
Geographic distribution	Local	141	26.8
	Within Sichuan	368	70.0
	Outside Sichuan	17	3.2
Operation Type	MVR	263	50.0
	AVR	91	17.3
	DVR	147	27.9
	TVR	12	2.3
	MVR + TVR	9	1.7
	DVR + TVR	4	0.8
Education level	Elementary School or below	119	22.6
	Junior High School	239	45.4
	Senior High School or Poly-technical School	130	24.8
	Junior College or above	38	7.2
Monthly family income	≤238 Dollar	100	19.0
	>238 Dollar	426	81.0

### Complications and patients satisfaction

According to the questionnaire, general complications such as gum bleeding, nasal cavity bleeding, profuse menstruation visible hematuria were observed in 19 patients, and no intracranial hemorrhage and other severe complications were observed. During this study, thrombus formation in the heart was judged through echocardiogram. We found no embolism or thrombus formation in this group of patients (Table 2).

**Table 2:** **Number of incidences of anticoagulation complications during this study (Unit: persons)**

Anticoagulation complications	Cases
Cerebral embolism	0
Intracranial hemorrhage	0
Thrombus formation	0
Gum bleeding	5
Nasal cavity bleeding	5
Profuse menstruation	6
Visible hematuria	3
Total	19

Traditional lab testing requires drawing 3 cm^3^ of venous blood while portable coagulometer only needs to draw about 0.01 cm^3^ of blood from the end of fingertip capillary, which may alleviate trauma and pain to the patient, and is thus more favorable to patients as compared with traditional blood collection. In the study, 409 persons (77.8%) preferred fingertip blood collection, 47 persons (8.9%) preferred vein blood collection and 70 persons (13.3%) considered both acceptable. In the study, we also found that the patients with anticoagulation therapy in China knew little about portable coagulometer and were worried about this “new type” of monitoring instrument. 485 persons (92.2%) have never heard of portable coagulometer; 432 persons (82.2%) were worried that the measured value of the instrument itself or the measured value obtained by them may not be accurate; 146 persons (27.8%) thought that the instrument was too expensive; 333 persons (63.3%) worried about the adjustment of treatment regimen after self-monitoring.

### Comparison of INR measurement values using the two testing methods

**Figure 1:**
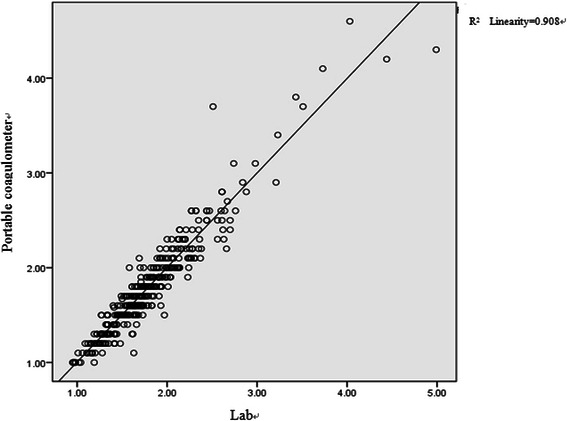
**INR linearity regression diagram of lab testing and CoaguChek XS testing.**

**Figure 2:**
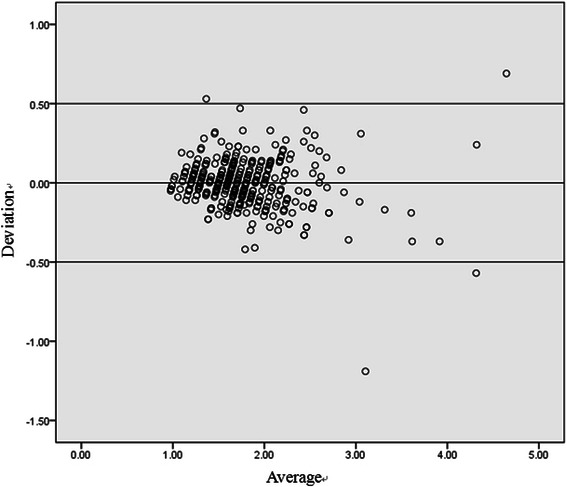
**Diagram of bias of the INR values from lab testing and from CoaguChek XS testing.**

Five hundred and twenty six patients were tested for 2079 times in total with CoaguChek XS portable coagulometer and traditional central lab automatic coagulometer in parallel. The results of linear regression analysis on the two groups of data are shown as the Figure 1. It is shown that the regression equation for measured value of CoaguChek XS portable coagulometer (y axis) and lab measured value (x axis) is y = 0.155 + 0.911x. Good correlation between the INR measured values of the two groups can be observed (r = 0.953, p < 0.05). Meanwhile, its bias can be observed with Bland-Altman diagram, as shown in Figure 2, the gap between the measured value from CoaguChek XS portable coagulometer and the measured value from traditional central lab is −0.033 ± 0.15732, proving good consistency between the two groups.

### Comparison of anticoagulation testing costs

The total expenses of the three groups of outpatient patients were counted (Table 3), in which, the expenses incurred to patients of Chengdu urban areas mainly came from registration fee and testing fee of the hospital with the average expenses of 9.83 ± 1.61 U.S dollar. The patients in other regions of Sichuan may afford more transportation fee and accommodation of 1–2 days, who were the majority of patients receiving anticoagulation outpatient diagnosis, accounting for 70%, with average expenses of 63.00 ± 45.53 U.S dollar. The number of patients from other provinces was small. They had to pay more for transportation and accommodation for re-examination visit to the outpatient with average expenses of 150.50 ± 69.60 U.S dollar. From the study, it can be seen that patients from various regions afforded different expenses for re-examination mainly due to the difference of transportation fee and accommodation fee varying with the distance while the expenses of diagnosis were basically the same. Pairwise comparisons among the three groups showed P < 0.05, all statistically significant. CoaguChek XS (Roche) portable coagulometer’s test strip is currently sold 9.5-14.3 dollar/strip. In China, Guangdong province and Jiangsu province have included the test strips into the scope of Medicare. By the comparison of the expenses required for each monitoring, we may see that the expenses for traditional monitoring methods of the Local Group of patients were slightly lower than those of the portable coagulometer monitoring, p > 0.05, the difference showed no statistically significant, while the expenses for traditional monitoring methods of the Sichuan Group (except Chengdu) and Outside-Sichuan Group of patients were obviously higher than those of the portable coagulometer monitoring, p < 0.05, the difference are statistically significant.

**Table 3:** **Comparison of re-examination expenses of the patients from different areas**

	Local group	Within Sichuan group	Outside Sichuan group
Number of patients (Persons)	141 (26.8%)	368 (70.0%)	17 (3.2%)
Testing fee (dollar)	1.74	1.74	1.74
Registration fee (dollar)	3.17	3.17	3.17
Transportation fee (dollar)	0-4.75 (1.97 ± 0.84)	15.84-126.73 (45.36 ± 21.46)	63.37-158.42 (100.63 ± 38.49)
Accommodation fee (dollar)	0	0-31.67 (14.97 ± 9.25)	15.84-63.37 (24.85 ± 13.64)
Others (dollar)	0-3.17 (1.72 ± 0.98)	0-9.50 (4.06 ± 2.74)	0-12.67 (5.68 ± 3.07)
Total (dollar)	4.91-12.67 (9.83 ± 1.62)	31.68-154.42 (63.00 ± 45.53)	95.05-221.78 (150.50 ± 68.60)

Patients from various regions consume almost the same amount of time in the hospital on the day of visit, mainly including: registration, queuing up for blood collection, waiting for reports, waiting for diagnosis, being diagnosed by doctors, paying fees, etc., about 3–5 hours in total. The difference in time mainly lies in the difference of time consumed in accommodation and in the journey (Table 4). For patients in urban areas of Chengdu, the time spent in the hospital accounts for a large proportion in the total time consumed. For patients outside Chengdu, the time consumed for accommodation and journey does so. Merely comparing the time required from blood collection to getting the result, lab monitoring usually takes 2–3 hours, about 4 hours to the maximum (155.17 ± 38.25 minutes), while in case of portable coagulometer monitoring, it only takes about 1 minute to complete the process from blood collection to getting results, and if adding the time for pre-warming of instrument, sterilization, fingertip puncturing, the total time is about 0.04 ± 0.03 hours. Conduct paired *T*-test for the two groups of data, p < 0.01, it is concluded that the time required for group one is obviously less than group two.

**Table 4:** **Comparison of time consumed for re-examination between patients adopting different monitoring methods**

	Region	Time consumed (hours)
	Local group	12.00-19.20 (12.48 ± 2.40)
Group 1	Within-Sichuan group	24.00-84.00 (47.76 ± 18.72)
	Outside-Sichuan group	48.00-108 (64.08 ± 13.92)
Group 2		0.03-0.08 (0.04 ± 0.03)

The annual average income per capita of Sichuan Province in 2012 was 6,007.76 dollars, namely monthly average income of 500.59 dollars or daily salary of 16.63 dollars. To set a uniformed standard, the economic benefits of Outside-Sichuan Group were evaluated using the average income of Sichuan. Considering the socially necessary labor time consumed, if the patient performs self-monitoring of anticoagulant therapy, it may save the time consumed in visits to the hospitals, of which the economic benefits are evaluated (Table 5). Time-saving effect of the Within-Sichuan Group and Outside-Sichuan Group was more substantial. Some patients need companion, so that such effect may be doubled.

**Table 5:** **Cost-benefit analysis of each testing in patient self-monitoring (Unit: U.S Dollar)**

	New time benefits	New cost benefits	New sum of time and cost benefits
Local group	8.65	−1.10	6.60
Within-Sichuan group	33.10	51.12	84.22
Outside-Sichuan group	44.41	138.61	183.03

## Discussion

During this study, general complications such as gum bleeding, nasal cavity bleeding, profuse menstruation visible hematuria were observed in 19 patients, and no intracranial hemorrhage and other severe complications were observed. In this experiment, we may see that the measured values of portable coagulometer were well consistent with those of the traditional lab methods. In our study, it can be easily seen that Within-Sichuan patients took a large percent in the out-patient patients, other expenses incurred to these patients for out-patient re-examination were much higher than the expenses required for blood collection testing, and the total expenses for each out-patient visit was also higher than that of portable coagulometer self-monitoring. Meanwhile, testing with portable coagulometer can obviously save the time consumed in each re-examination visit of each anticoagulation patient.

Traditional lab monitoring of INR is usually used to test the accuracy of the measured value of other INR testing equipment as a gold standard [18]. In our study, results of CoaguChek XS monitor were consistent with relevant overseas and domestic reports [15,18–25]. There were also other reports that when INR value was greater than 3.5, the measured value of CoaguChek XS portable coagulometer will be higher than the lab measured value, showing decreased accuracy. In this experiment, since all the patients adopted low-intensity anticoagulant therapy, INR was maintained at a low level; therefore few data have INR in the measured value greater than 3.5, thus disabling a systematic evaluation of the correlation of the two [20]. However, when INR is maintained at a low level, the measured value of portable coagulometer is convincing and thus suitable to the monitoring and management of patients in China.

For the patient as an individual, the economic benefit comparison varies greatly among different countries. Such gap mainly comes from the difference of Medicare system [26]. In some countries with consummated Medicare, portable coagulometer and the test strips have been listed as items of medical insurance, thus alleviating the burden of the patients. In the case of China presently, patients in most areas shall pay relevant expenses on their own. In a long run, the life of portable coagulometer is 30,000 times with zero maintenance cost and thus eliminating the need of frequent replacement of instrument. Even if the initial costs such as procurement of instrument are included, the average direct cost of portable coagulometer self-monitoring is obviously lower than that of the traditional monitoring methods. Testing with portable coagulometer can obviously save the time consumed in each re-examination visit of each anticoagulation patient. Meanwhile, it also reduced the workload of the out-patient doctors. The time saved, as converted into economic benefits may further prove the cost advantages of patient self-monitoring.

Presently, in some developed western countries, most patients have adopted portable coagulometer to perform self-monitoring (PST: patients measure their INR values by themselves, and professional doctors or pharmacists gave the adjusted regimen for warfarin administration) and self-management (PSM: patients measure their INR values by themselves, and adjusted their warfarin administration regimen by themselves) of oral anticoagulant treatment, and also accumulated lots of experience [13,27,28]. In China, the portable coagulometer is rather less popular. The main reasons include:

- Patients are concerned about short-term cost while ignoring long-term cost. One unit of CoaguChek XS portable coagulometer costs 1267. 33 dollars, which is a large expense for many Chinese families. The initial cost is obviously higher than that of the traditional monitoring method.
- Not well-informed of the self-monitoring of anticoagulant therapy. From the questionnaire, it was shown that patients receiving anticoagulation treatment in China knew too little about portable coagulometer and most patients have never heard of portable coagulometer, and therefore balked at using self-monitoring and management.
- In many remote areas, patients who only have low level of education fail to complete self-monitoring of anticoagulant therapy.
- In most areas in China, such item has not been covered by Medicare, indirectly increases expenses afforded by the patients.

With the economic development of China, especially the increase of income of rural patients, the price of portable coagulometer is no longer prohibitive to the patients. If the importance of anticoagulant therapy and the advantages of self-monitoring of anticoagulant therapy are further informed to the patients, portable coagulometer may become the first choice for anticoagulation patients, enjoying a prospect of wide application. Due to the unreasonable distribution of medical resources in China, the traditional monitoring methods brought about heavy economic burden to patients in remote areas. To alleviate such burden, these patients lengthen their re-examination intervals on their own will, thus becoming less compliant, and more vulnerable to complications. Therefore, such kinds of patients are the most ideal group for conducting self-monitoring of anticoagulant therapy with portable coagulometer.

### Limitation

These findings are subject to certain limitations. First, we followed up the patients for a short time period, and the study methods were found to be less than ideal. Second, we only describe the present situation of anticoagulation monitoring in patients after mechanical heart valve replacement in China and point to the failure of regular monitoring in a high percentage of patients. The final limitation of this study is that the economic evaluation is simple.

## Conclusion

The measured value of CoaguChek XS INR monitor is accurate, showing good consistency and stability with the results of traditional lab testing, while saving cost and time, improving monitoring frequency, reducing the incidence of anticoagulation complications, improving the life quality of patients and showing good prospect. It is advisable to intensify the self-monitoring and management of patient and improve the knowledge of patients in this regard. Meanwhile state support such as inclusion into medical insurance is also required for its further popularization.
